# Identification of PLATZ genes in *Malus* and expression characteristics of MdPLATZs in response to drought and ABA stresses

**DOI:** 10.3389/fpls.2022.1109784

**Published:** 2023-01-18

**Authors:** Yaqiang Sun, Yunxiao Liu, Jiakai Liang, Jiawei Luo, Fan Yang, Peien Feng, Hanyu Wang, Bocheng Guo, Fengwang Ma, Tao Zhao

**Affiliations:** ^1^ State Key Laboratory of Crop Stress Biology for Arid Areas, College of Horticulture, Northwest A&F University, Yangling, Shaanxi, China; ^2^ Shaanxi Key Laboratory of Apple, College of Horticulture, Northwest A&F University, Yangling, Shaanxi, China; ^3^ Xinjiang Production & Construction Corps Key Laboratory of Protection and Utilization of Biological Resources in Tarim Basin, Tarim University, Alar, Xinjiang, China

**Keywords:** PLATZ, Rosaceae species, *Malus*, drought stress, co-expression network

## Abstract

Plant AT-rich sequences and zinc-binding proteins (PLATZ) play crucial roles in response to environmental stresses. Nevertheless, PLATZ gene family has not been systemically studied in Rosaceae species, such as in apple, pear, peach, or strawberry. In this study, a total of 134 PLATZ proteins were identified from nine Rosaceae genomes and were classified into seven phylogenetic groups. Subsequently, the chromosomal localization, duplication, and collinearity relationship for apple PLATZ genes were investigated, and segmental duplication is a major driving-force in the expansion of PLATZ in *Malus*. Expression profiles analysis showed that PLATZs had distinct expression patterns in different tissues, and multiple genes were significantly changed after drought and ABA treatments. Furthermore, the co-expression network combined with RNA-seq data showed that PLATZ might be involved in drought stress by regulating ABA signaling pathway. In summary, this study is the first in-depth and systematic identification of PLATZ gene family in Rosaceae species, especially for apple, and provided specific PLATZ gene resource for further functional research in response to abiotic stress.

## Introduction

Drought stress is a main limiting environmental factor for plants growth and crop yield, which could affect plant physiological and biochemical metabolism, eventually leading to food and societal problems (Ergo et al., 2018; Rahmati et al., 2018). To date, numerous studies have been performed to investigate the underlying mechanisms of drought stresses in plant. To cope with drought stress, plants has evolved a series of physiological adaptations, such as changes of the morphological characteristics, osmotic regulation, and endogenous hormones levels (Silva et al., 2010; Bhargava and Sawant, 2013, Liu et al., 2014).

Transcription factors (TF) play essential roles in multiple physiological processes in plants, in which it could recognize and bind to *cis*-acting elements in the promoter regions of target genes to regulate downstream signaling pathways (Mitchell and Tjian, 1989; Ptashne and Gann, 1997; Riechmann et al., 2000). At present, more than fifty TFs families have been identified in plants. The zinc finger transcription factor is one of the largest TF families in plants, including ERF, PHD, PLATZ, ZFP, TZF, and WRKY (Zhang et al., 2022). Previous studies have found that they were involved in various environmental stresses, especially drought stress (Han et al., 2019; Sun et al., 2019). Among them, C2H2 zinc finger proteins (ZFPs) and CCCH-tandem zinc finger proteins (TZFs) have been found to be participated in plant development and abiotic stress responses. For example, the zinc finger protein TaZFP1B increased drought tolerance in transgenic wheat plants by regulating the expression of oxidative stress responsive genes and reducing the ROS accumulation (Cheuk et al., 2020). Similarly, the rice *OsTZF5* overexpression lines showed increased drought tolerance with high single-plant level and per unit grain yield compared with the NT plants (Selvaraj et al., 2020). Besides, other zinc finger proteins such as *OsMSR15*, *OsDRZ1*, *AtZAT18*, *MtPHD6*, and *CmBBX19*, were also identified as key regulators of plant drought tolerance (Zhang et al., 2016; Yin et al., 2017; Yuan et al., 2018; Quan et al., 2019; Xu et al., 2020).

The plant AT-rich sequences and zinc-binding (PLATZ) proteins belong to a novel class of plant specific zinc finger transcription factor, with two distant-conserved domains (Nagano et al., 2001). PLATZ genes play important roles in several biological processes, including plant growth and development (Gonzalez-Morales et al., 2016; Kim et al., 2018; Zenda, 2019; Jun et al., 2020; Liu et al., 2020; Dong et al., 2021). In rice, *GL6* as a rice grain length QTL, positively affected grain length and yield by regulating cell division (Wang et al., 2019). *ZmPLATZ12*, specifically expressed in endosperm starchy cells in maize (*Zea mays*), can affect endosperm development and storage reserve, by interacting with RPC53 and TFC1 (Li et al., 2017). ORESARA15 (ORE15), a PLATZ-type transcriptional activator in *Arabidopsis*, positively regulates the expression of leaf size related- and senescence related-genes (Kim et al., 2018). Furthermore, PLATZ proteins in plants are also involved in response to abiotic stresses (Zhang et al., 2018; Blair et al., 2019; Zenda, 2019; Liu et al., 2020; Dong et al., 2021). For example, the PLATZ family member AIN1 participated in the regulation of ROS homeostasis in *Arabidopsis* after ABA treatment (Dong et al., 2021). Overexpression of soybean *GmPLATZ1* delayed germination and cotyledon development in transgenic *Arabidopsis* under ABA and osmotic stresses (So et al., 2015). In *Glycine max* and *Zea mays*, the expressions of *GmPLATZ1*, *GmPLATZ17*, and *ZmPLATZ* were induced under drought treatment (So et al., 2015; Zenda, 2019; Zhao et al., 2022).

Apple (*Malus domestica*), a representative species of the Rosaceae family, is one of the most economically important fruit crops in the world. The unique light environment and temperate climate of China’s Loess Plateau, making it become one of the most productive apple cultivation areas in the world (Yan et al., 2015). However, a limited water supply threatens the sustainable production of apple in this region (Wang et al., 2018). PLATZ genes play vital roles in resistance to abiotic stress in plants. However, the PLATZ gene family has not been identified and functional characterized in Rosaceae species. Here we present a genome-wide analysis of the PLATZ proteins in Rosaceae species, especially in *Malus*. The expression patterns of apple PLATZ genes in different tissues were investigated using public transcriptomic data, the expressions of *MdPLATZ* genes in apple roots under abiotic stress in *Malus* were estimated by quantitative real-time PCR. In addition, the co-expression network of PLATZ genes was constructed in *Malus*. In general, our findings provide a valuable insight into the evolutionary history of PLATZ genes in Rosaceae species and will be useful for future studies dissecting the regulatory mechanisms of PLATZ genes.

## Materials and methods

### Identification of PLATZ genes in the apple genome

Genome annotations of apple genomes (*M. domestica* cv. Golden delicious (Md), *M. domestica* cv. Hanfu (Mdhf), *M. domestica* cv. Gala (Mdg), *M. sieversii* (Msi), *M. baccata* (Mb), and *M. sylvestris* (Msy)) and three other Rosaceae species genomes *Fragaria vesca* v4.0 (FvesH4), *Prunus persica* (ppe), and *Pyrus communis* L. (Pycom) were downloaded from the Genome Database for Rosaceae (https://www.rosaceae.org/) (Jung et al., 2019). The genome annotations of *Arabidopsis thaliana* and *Oryza sativa* were retrieved from TAIR (https://www.arabidopsis.org/) and Phytozome (https://phytozome-next.jgi.doe.gov/), respectively. The hidden Markov model (HMM) profile of the PLATZ domain (PF04640) was downloaded from the Pfam database (http://pfam.xfam.org/) and then was exploited for the identification of the PLATZ genes in the species described above. Putative PLATZ gene sequences were submitted to Simple Modular Architecture Research Tool (SMART) website (http://smart.embl-heidelberg.de/) to check the completeness of the PLATZ conserved domain (Finn et al., 2011). The physicochemical parameters of each gene were calculated using the ExPASy (https://web.expasy.org/protparam/), including molecular weight (MW) and theoretical isoelectric point (pI) tool. In addition, the subcellular localization of PLATZs was investigated using Cell-PLoc 2.0 (Chou and Shen, 2008).

### Phylogenetic relationship and conserved motif analysis of PLATZ genes

Multiple sequence alignment was conducted using the MUSCLE tool integrated in MEGA6.0, with default parameter (Larkin et al., 2007). The maximum likelihood (ML) tree was constructed based on the full-length protein sequences using the MEGA software with a bootstrap of 1000 replications. The tree file was visualized using FigTree v1.4.2 software, and then was modified using Interactive Tree of Life (iTOL) (http://itol.embl.de/). To obtain the conserved motifs, the PLATZ proteins in apple were analyzed using Multiple Em for Motif Elicitation (MEME) Version 5.4.1 (http://meme-suite.org/meme/tools/meme) with the default parameters. The visualization of PLATZ domains was performed using TBtools v1.098 (Chen et al., 2020). The exon/intron structures of PLATZ family genes in apple were analyzed by the Gene Structure Display Server online program with default parameters (GSDS: http://gsds.cbi.pku.edu.ch) (Hu et al., 2015). Finally, the upstream 2 kb genomic DNA sequences of all *MdPLATZ* genes were extracted, and then submitted to PlantCARE (http://bioinformatics.psb.ugent.be/webtools/plantcare/html/) to predict the *cis*-acting elements in the promoter region.

### Gene duplication and evolutionary rate analysis

MCScanX was applied to identify tandem and segmental duplications of PLATZ genes among *Malus* species, and the syntenic relationships were plotted using the Circos software (http://circos.ca/) (Wang et al., 2012). The rate of Ka (nonsynonymous substitutions site)/Ks (synonymous substitutions site) was applied to assess the evolutionary rate of PLATZ genes, which was calculated by TBtools (v1.098).

### Expression pattern analysis using RNA-seq data

The RNA-seq data of PLATZ genes in 16 different tissues of the *Malus* was downloaded from the NCBI database (https://www.ncbi.nlm.nih.gov), including root, stem, leaf, tree shoot apex, dormant buds, flower, stigma, style, ovary, filament, anther, pollen, petals, sepal, receptacle, and fruit, the registration numbers were listed in **Supplementary Table 3**. The FPKM (fragments per kilobase of transcript per million fragments mapped reads) of all PLATZ genes were calculated using Hisat2 and Stringtie software (Pertea et al., 2016)(**Supplementary Table 4**). Then, differentially expressed genes were identified with the following threshold values: log2fold change ≥1, FDR (false discovery rate) ≤ 0.05. In addition, the Pearson correlation of the expressions of *MdPLATZ* genes in different tissues were calculated using GraphPad Prism tool, with *p* < 0.01 considered as significantly different among different samples.

### Plant material and treatment

The tissue-cultured *M. domestica* cv. Golden delicious plants were initially grown on MS (Murashige and Skoog) agar media. After rooting on MS agar media, the apple seedlings were transferred to small plastic pots containing a mixture of soil/perlite and then were cultured in a climate chamber with a 16 h light/8 h dark photoperiod and 55-65% relative humidity at 24°C. Stress treatments were performed to the apple seedlings as described previously (Zhao et al., 2020), including drought and ABA. For the drought treatment, the apple plants roots were collected at 0, 12, 24, 72, and 120 hours after drought stress. For the abscisic acid (ABA) treatment, the apples roots were sampled at 0, 3, 9, 12, and 24 hours after treatments. All the samples were snap-frozen in liquid nitrogen and stored at -80°C for RNA extraction. Three biological replicates were collected at each time point. The correlation coefficients among *MdPLATZ* genes under drought and ABA treatments were calculated using the Pearson correlation in GraphPad Prism tool, with *p* ≤ 0.01 considered as significantly different among different samples.

### RNA isolation and expression profiling analysis

The experiment of qRT-PCR was applied to detect the expression patterns of the *MdPLATZ* genes in roots after drought and ABA treatments. Total RNA was isolated using the RNAprep Pure Plant Kit (TIANGEN, Beijing, China) according to the manufacturer’s instructions. cDNA was synthesized from total RNA using the HiScript II 1st Strand cDNA Synthesis Kit (+gDNA wiper) (Vazyme, Nanjing, China), and used as the qRT-PCR template. The primer pairs used in this study were listed in **Supplementary Table 5**. The *MdEF-1α* gene was used as the reference gene to normalize the gene expression in each qRT-PCR experiment. The relative expression of each gene was calculated based on the 2^-△△CT^ method (Livak and Schmittgen, 2001). All statistical analyses were performed by *t* tests, with *p* ≤ 0.05 considered as significantly different among different groups.

### Subcellular localization of *MdPLATZ* proteins

The coding sequences of *MdPLATZ2*, *MdPLATZ10*, and *MdPLATZ11* were cloned by the designed primers (**Supplementary Table 5**). Then the sequences (without the termination codons) were individually cloned into the pRI101-GFP vector. Subsequently, validated vectors were individually transformed into *Nicotiana benthamiana* leaves with nuclear localization plasmid pHBT-NLS-mCherry and *35S::MdPLATZ2/10/11::GFP* vectors. The empty vector was used as a control. After incubation in the dark place for 48 h, the fluorescence signals in the leaves of the transfected plants were observed under the fluorescence microscope (Leica SP5).

### Co-expression network analysis of PLATZ family genes

The co-expressed genes of apple PLATZ genes were predicted using the AppleMDO database (http://bioinformatics.cau.edu.cn/AppleMDO/) (Da et al., 2019). Subsequently, the genes were predicted to be correlated with 17 MdPLATZ genes were identified and then applied to construct the co-expression network using Gephi v0.9.2 (Bastian et al., 2009). Gene Ontology (GO) and Kyoto encyclopedia of gene and genome (KEGG) enrichment analysis was performed among these genes and displayed using agriGO v2.0 (Tian et al., 2017) and KOBAS tool (Bu et al., 2021).

## Results

### Identification of the PLATZ family genes in *Malus* species

Using the HMM profile of the PLATZ domain (PF04640), a total of 104 candidate PLATZ proteins were identified from six apple genomes. A total of 14, 17, 17, 19, 19, and 18 PLATZ proteins were identified in the genomes of Mdhf, Md, Mdg, Msi, Msy, and Mb, respectively (**Supplementary Figure 1**; **Supplementary Table 1**). The lengths of the PLATZ CDS regions varied from 399 bp (*Mdgplatz15*) to 2679 bp (*Mbplatz6*), and the putative proteins were predicted to encode 133 to 893 amino acids in length. The MW for the predicted PLATZ proteins ranged from 15.31499 kDa to 99.11895 kDa, and the isoelectric points ranged from 6.1 to 9.94. Moreover, the subcellular localization of PLATZs was predicted by Cell-PLoc, and most PLATZ proteins were located in the cell nucleus (**Supplementary Table 1**).

### Phylogenetic analysis of the PLATZ genes

**Figure 1:**
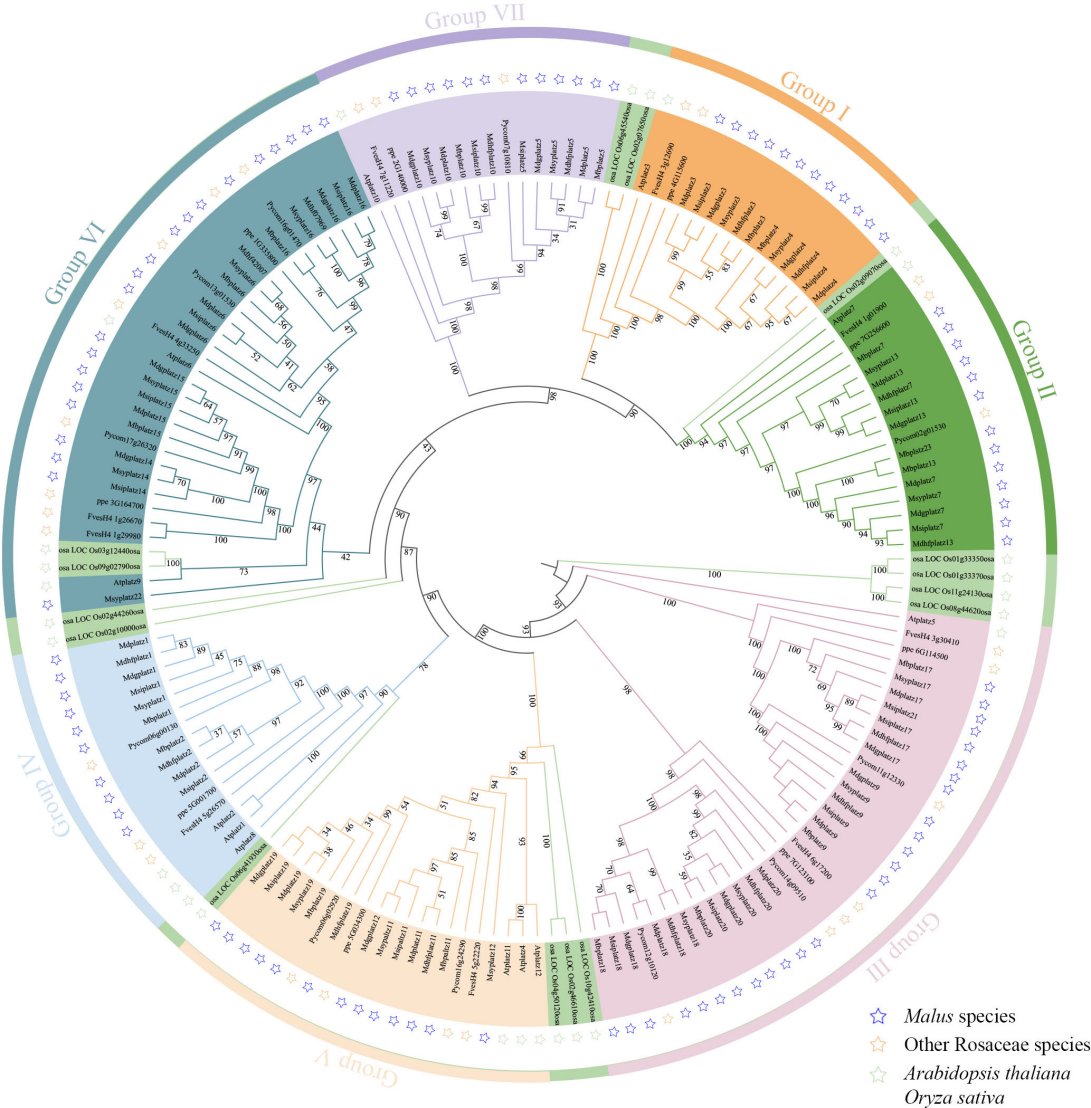
The maximum-likelihood phylogenetic tree of the PLATZ genes. I-VII represent seven groups marked in different colors. The blue, orange, and green pentacles represent the PLATZ genes from *Malus*, other Rosaceae species (*Fragaria vesca* v4.0, *Prunus persica*, and *Pyrus communis* L.), and model plants (*A. thaliana* and *O. sativa*), respectively.

To understand the evolutionary relationships of PLATZ genes, a phylogenetic tree based on full-length protein sequences was constructed by the maximum likelihood method. A total of 161 PLATZ homologous genes from 11 representative species, including 6 *Malus* species (104 PLATZ proteins), *Fragaria vesca* v4.0 (10 PLATZ proteins), *Prunus persica* (9 PLATZ proteins), *Pyrus communis* L. (11 PLATZ proteins), *A. thaliana* (12 PLATZ proteins), and *Oryza sativa* (15 PLATZ proteins). As shown in **Figure 1**, the PLATZ genes were phylogenetically categorized into seven groups (I to VII), In detail, 17, 18, 32, 14, 16, 25, and 16 PLATZ genes were identified from group I to group VII, respectively. The PLATZ genes in *O. sativa* were separated from Rosaceae species and *A. thaliana*, implying that PLATZs in apple were more closely related to AtPLATZs. In addition, most *Pyrus* PLATZs and *Malus* PLATZs formed sister clades (**Figure 1**; **Supplementary Figure 2**), which suggested that the PLATZ genes in pear and *Malus* had a closer evolutionary relationship.

### Gene structural characterization and conserved motifs among *Malus* PLATZ genes

**Figure 2:**
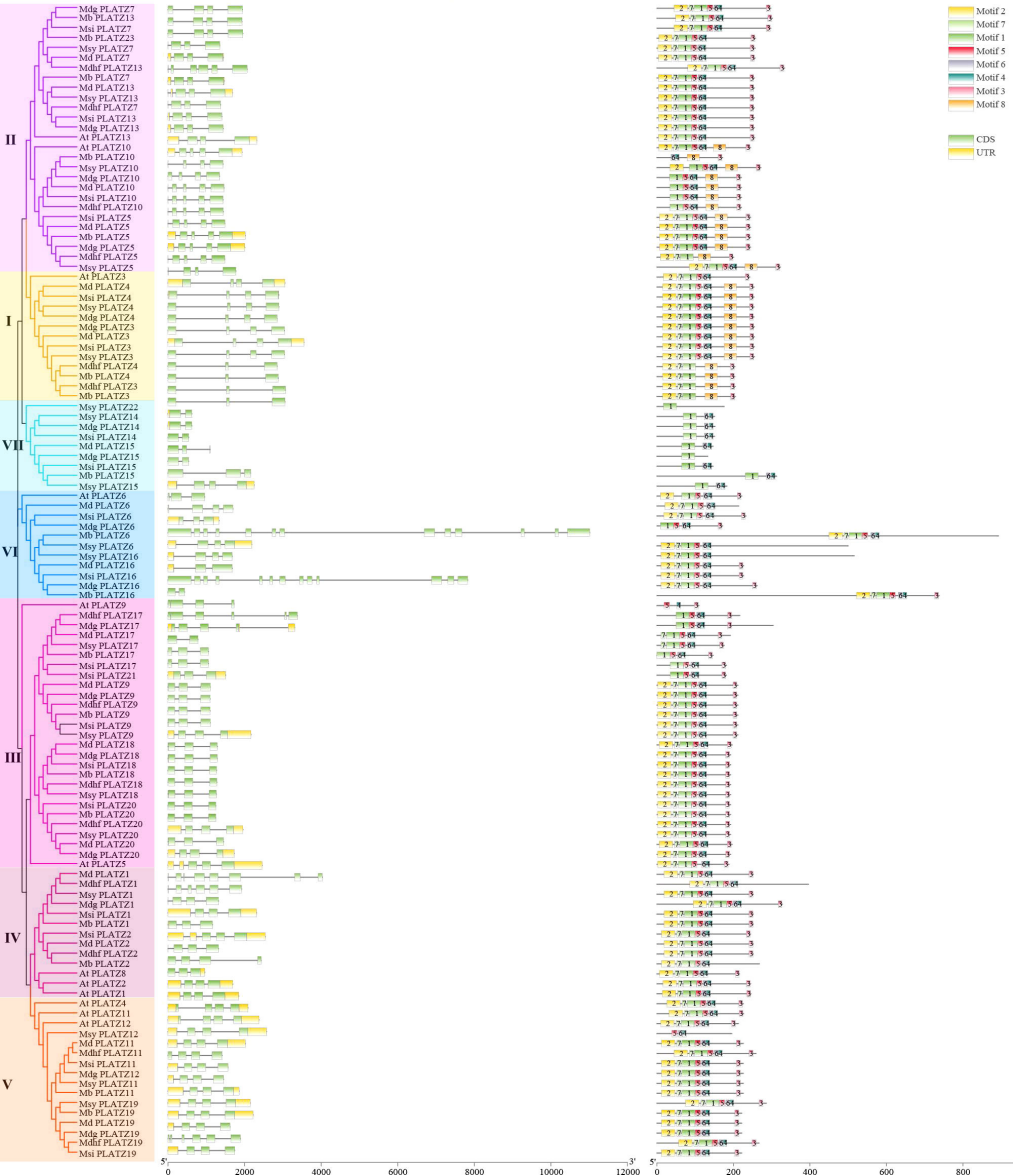
Distribution of conserved motifs and exon–intron structures of the PLATZ family genes in *Malus* species. The phylogenetic tree was constructed by MEGA-6, using full-length amino acid sequences (1000 Bootstrap replicates and LG substitution model). Different colors in the branches indicate different groups. Green and yellow boxes indicate the coding sequences and the untranslated regions, black lines represent the introns. A total of 8 motifs (motif 1-8) were predicted by MEME tool, and the boxes with different colors indicate different motifs.

The gene structure similarities could provide a clue of the gene family evolution history. Among 104 apple PLATZs, most gene members in the same groups shared similar exon/intron structures in terms of exon/intron number and exon length (**Figure 2**). For example, the PLATZ genes in subfamily III contain three exons, while those in subfamily V mostly have four exons, except for *Mdhfplatz19*. Interestingly, the gene structure appeared to be more variable in subfamilies VI, in which the number and length of the exons were remarkably distinct. The number of introns in all *Malus* PLATZ genes ranged from 1 (*Mbplatz16*) to 12 (*Mbplatz6*), indicating that some introns in PLATZ genes were lost during evolution. The numbers of exons in the same apple species varied greatly, ranging from 2 (*Mdgplatz14*) to 13 (*Mdgplatz16*), indicating that they might be involved in different biological processes. In addition, the PLATZ homologous genes among different apple species exhibited slight variation. For apple PLATZ 10, exon lengths were different between Mdg and Msy.

Exploring the motifs distribution is meaningful to understanding gene function and their evolutionary history (Han et al., 2022). We further investigated the conserved motifs of PLATZ genes, and 8 distinct motifs were identified in apple by the MEME program, designated motifs 1-8 (**Supplementary Figure 3**). The number and relative position of motifs in PLATZ members were relatively conserved in *Malus*. Among them, most apple PLATZ members contained motif 2, motif 7, motif 1, motif 5, motif 6, and motif 4. As expected, most members clustered into the same groups contained common motif structures. Interestingly, some motifs were found to be specific in groups. For instance, motif 8 was present in the genes of group I and part of group II. Motif 1, motif 6, and motif 4 were specific for group VII. These results indicated that PLATZ proteins in these groups might have different biological functions. Additionally, we found motif 5, motif 6, and motif 4 were specific to *Msyplatz12*, and motif 1 was specific to *Msyplatz22* (**Figure 2**). In summary, most genes clustered into the same groups had similar gene structures and conserved motifs, suggesting that proteins from the same groups may have similar biological functions. The divergences in the number of motifs and exons in different groups, indicating that these genes were involved in multiple biological processes.

### Duplications and collinearity analysis of PLATZ genes in *Malus*

**Figure 3:**
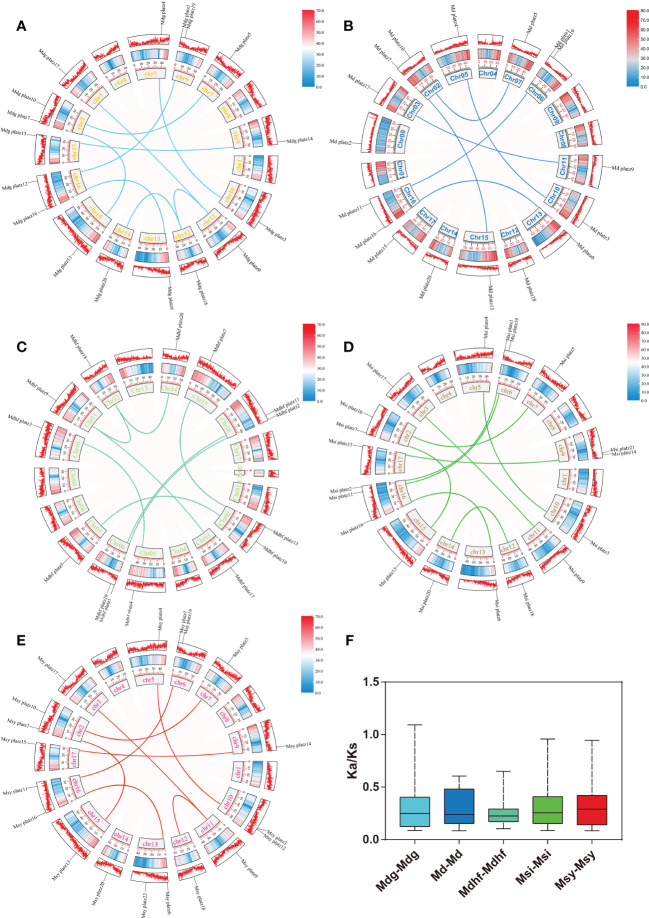
Synteny relationships of the PLATZ genes in *Malus* species. **(A–E)** The chromosomal distribution and intra-genomic collinearity of the Mdg, Md, Mdhf, Msi, and Msy genomes. Different homologous gene pairs are connected by different colored lines in five *Malus* species. The light red ribbons represent syntenic blocks in the Malus genome, and the segmental duplication events are marked in different colors. **(F)** Boxplot showing the statistics of Ka/Ks values of the gene pairs in five apple species.

**Figure 4:**
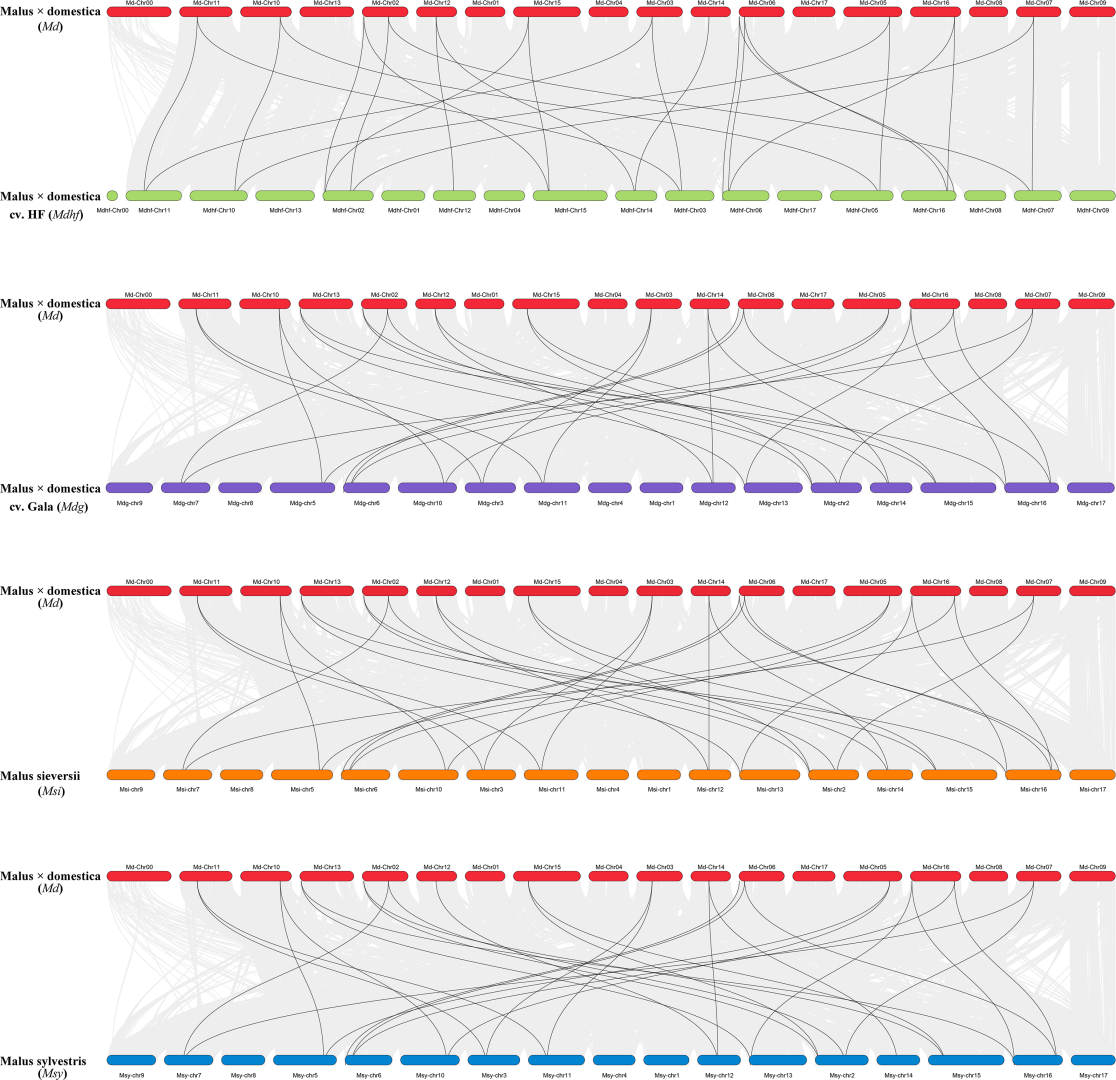
Collinear analysis between Md and other four *Malus* species (Mdhf, Mdg, Msi, and Msy). The position distribution of the PLATZ gene was marked with boxes on 17 chromosomes. Collinear gene pairs in apple genomes were linked by gray lines, the black lines inter-connectted the collinear gene pairs of the PLATZ gene family.

The identified 86 *Malus* PLATZs were mapped and positioned on 17 chromosomes (**Figure 3**), except for Mb PLATZ genes (without chromosome information). There was no PLATZ gene found on the Chromosome 1, 4, and 8. Furthermore, in order to explore the gene duplication and the diversity of PLATZs genes in *Malus* species, both intra- and intergenomic syntenic analyses of the PLATZ genes were carried out among the five *Malus* species. A total of 40 PLATZ genes showed a syntenic relationship within *Malus* species. Specifically, there were nine pairs of segmental duplications on the chromosomes of the Mdg genome (**Figure 3A**), and most of the duplicated PLATZ genes belongs to the same groups. In the genomes of Md and Msi, six and nine segmental duplication gene pairs were found of PLATZ genes, respectively (**Figures 3B-D**). Eight segmental duplication gene pairs were found in *MdhfPLATZ* and *MsyPLATZ* gene families, and one tandem duplication event occurred between *MsyPLATZ3* and *MsyPLATZ12* on chromosome 10 (**Figures 3C-E**). In addition, we calculated the *Ka* and *Ks* values of gene pairs within five genomes, and all genome Ka/Ks ratios were lower than 1, indicating that these gene pairs had experienced purifying selection (**Figure 3F**). To further analyze the evolutionary process of apple PLATZ genes, a comparative analysis of genome collinearity was conducted between Md and other four *Malus* (Mdhf, Mdg, Msi, and Msy) species. Notably, the results showed that the homologous genes between Md and other four Malus were tightly linked, with 30 homologous gene pairs in Msi, followed by Mdg (29 homologous gene pairs), Msy (28 homologous gene pairs), and Mdhf (25 homologous gene pairs) (**Figure 4**).

### Tissue expression patterns of MdPLATZ genes in *Malus domestica*

**Figure 5:**
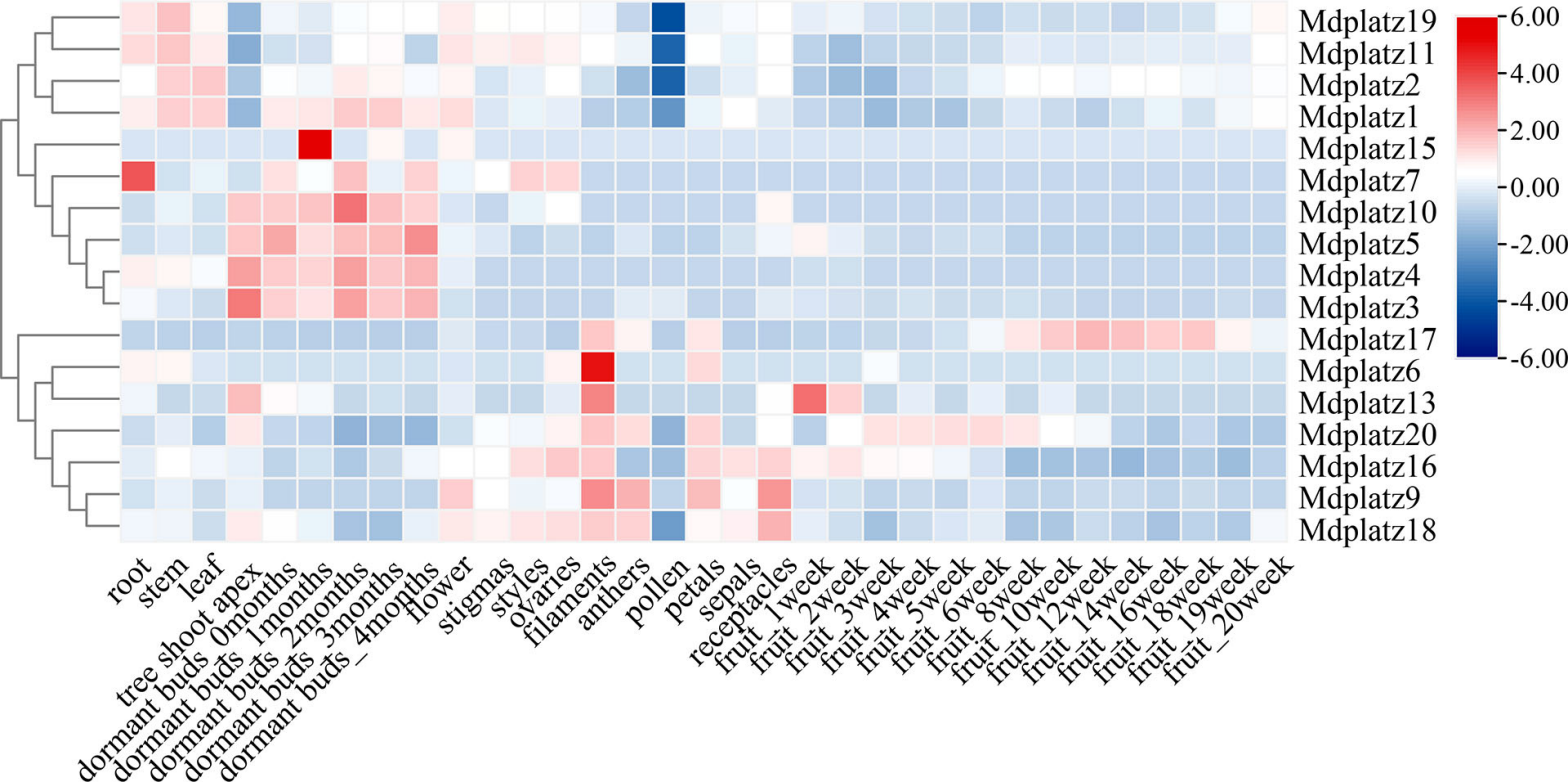
Expression of MdPLATZ genes in different tissues including root, stem, leaf, tree shoot apex, dormant buds, flower, stigma, style, ovary, filament, anther, pollen, petals, sepal, receptacle, and fruit.

To investigate the spatiotemporal expression patterns of the *MdPLATZ* gene family members, the transcriptomic data obtained from 16 different apple tissues were used (see materials and methods, **Figure 5**; **Supplementary Table 2**). In general, the expression patterns of *MdPLATZ* genes varied greatly in different tissues, indicating that they might be involved in multiple biological functions in *M. domestica*. Most *MdPLATZs* exhibited higher expression in shoot apex, filament, and bud, but lower expression in pollen (**Figure 5**). Seven *MdPLATZ* (*Mdplatz1*, *Mdplatz2*, *Mdplatz4*, *Mdplatz6*, *Mdplatz7*, *Mdplatz11*, and *Mdplatz19*) genes displayed higher expression tendencies in root, leaf, and stem tissues. Interestingly, eight genes (*Mdplatz3*, *Mdplatz4*, *Mdplatz5*, *Mdplatz7*, *Mdplatz10*, *Mdplatz11*, *Mdplatz17*, and *Mdplatz20*) showed distinct expression patterns in bud and fruit tissues collected at different developmental stages.

The correlation analyses of PLATZ genes were conducted, and the results suggested that multiple *MdPLATZ* genes were significantly correlated with each other (**Supplementary Figure 4**). Specifically, *Mdplatz2* was significantly positively correlated with *Mdplatz1*, *Mdplatz11*, and *Mdplatz19* (*p* < 0.01), and significantly negatively correlated with *Mdplatz20* (*p* < 0.01). *Mdplatz10* was positively correlated with *Mdplatz3*, *Mdplatz4*, and *Mdplatz5* (*p* < 0.01), and was negatively correlated with *Mdplatz11* (*p* < 0.01). In addition, *Mdplatz6*, *Mdplatz9*, *Mdplatz13*, *Mdplatz16*, *Mdplatz18*, and *Mdplatz20* were tightly correlated with each other, indicating that these genes might have synergistic relationships in response to external stresses. *Mdplatz7*, *Mdplatz15*, and *Mdplatz17* were weakly correlated with other *MdPLATZ* genes, indicating that those genes may participated in distinct functional modules during development (**Supplementary Figure 4**).

### Expression patterns of MdPLATZ genes under drought and ABA treatment

**Figure 6:**
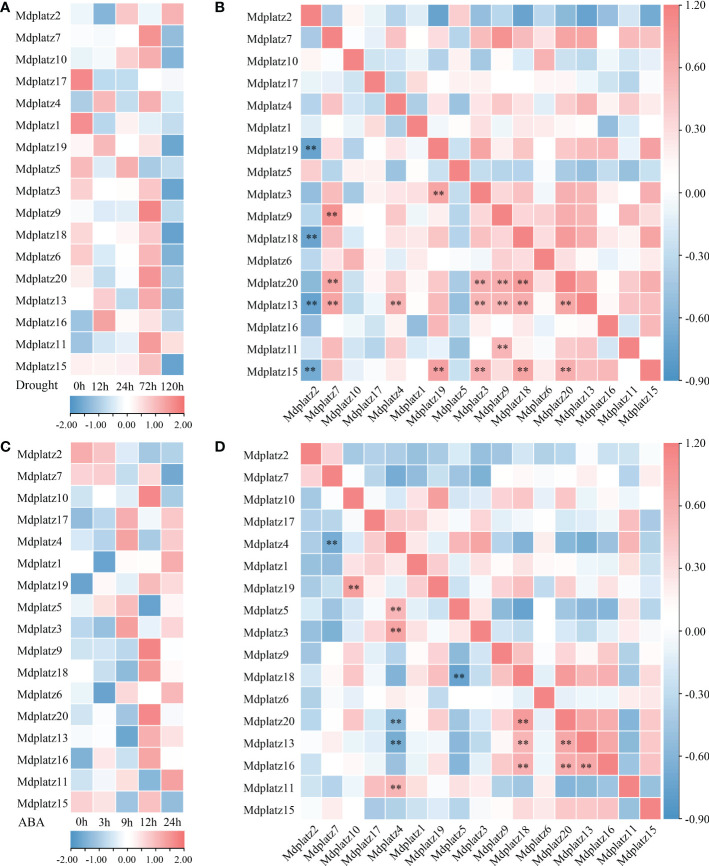
Expression analysis of MdPLATZs under drought and ABA treatments. **(A)** The expression levels of MdPLATZs in apple roots under drought stress. **(B)** Pearson’s correlation of the expression patterns of 17 *MdPLATZ* genes under drought stress. **(C)** The expression levels of MdPLATZs in apple roots under ABA treatment. **(D)** Pearson’s correlation of expression patterns in 17 *MdPLATZ* genes under ABA treatment. **indicates a significance correlation at the levels of p < 0.01.

The promoter sequences (upstream 2000 bp) of PLATZ genes were queried at PlantCARE, the results showed that most *MdPLATZ*s contained ABA- and drought-responsive *cis*-acting regulatory elements (**Supplementary Table 3**). Subsequently, the qRT–PCR was performed to investigate the expression patterns of *MdPLATZ* genes in *Md* roots under drought and ABA treatments. The results showed that 10 *MdPLATZ* genes were differentially expressed after drought stress. Four genes, *Mdplatz9*, *Mdplatz10*, *Mdplatz11*, and *Mdplatz13*, were significantly upregulated in roots, while six PLATZ genes, containing *Mdplatz3*, *Mdplatz4*, *Mdplatz17*, *Mdplatz18*, *Mdplatz1*, and *Mdplatz19*, were significantly downregulated under drought stress (**Figure 6A**). The correlation analysis results suggested that multiple *MdPLATZ* genes were significantly correlated with each other under drought stress (**Figure 6B**). As a result, *Mdplatz7* was positively correlated with *Mdplatz13* (group II) and *Mdplatz9 & 20* (group III) (*p* < 0.01), whereas *Mdplatz2* was negatively correlated with *Mdplatz15* (group II), *Mdplatz19*, and *Mdplatz18 & 20* (group III) (*p* < 0.01). Furthermore, the *Mdplatz13*, *Mdplatz6 & 16* (group VI), and *Mdplatz9 & 18 & 20* (group III) genes showed positively correlation relationship with each other, implying that these genes may be regulated cooperatively and may have relevant functional associations during drought stress (**Figure 6B**).

To understand the roles of PLATZ genes in apple roots under ABA treatment, the qRT-PCR was conducted, and the results showed that eight PLATZ genes, such as *Mdplatz1*, *4*, *13*, *6*, and *16* (group VI), and *Mdplatz9*, *17*, and *19* (group III) were significantly upregulated after ABA treatment. Conversely, *Mdplatz2*, *Mdplatz1*, *Mdplatz13*, and *Mdplatz18* showed a downregulated tendency under ABA treatment (**Figure 6C**). Subsequently, the correlation analysis results showed that the expression patterns of some *MdPLATZ* genes were significantly correlated under ABA treatment (**Figure 6D**). The *Mdplztz7* and *Mdplztz4* showed negatively correlated relationship (*p* < 0.01), while *Mdplztz10* and *Mdplztz19* showed a significant positive correlation relationship (*p* < 0.01). *Mdplatz4* was positively correlated with *Mdplatz3*, *Mdplztz5*, and *Mdplztz11*, but negatively correlated with *Mdplztz13* and *Mdplatz20* (*p* < 0.01). Similarly, *Mdplatz9*, *Mdplatz13*, *Mdplatz16*, *Mdplatz18*, and *Mdplatz20* were positive correlated with each other (**Figure 6D**).

### Subcellular localization of MdPLATZs in *Malus domestica*

**Figure 7:**
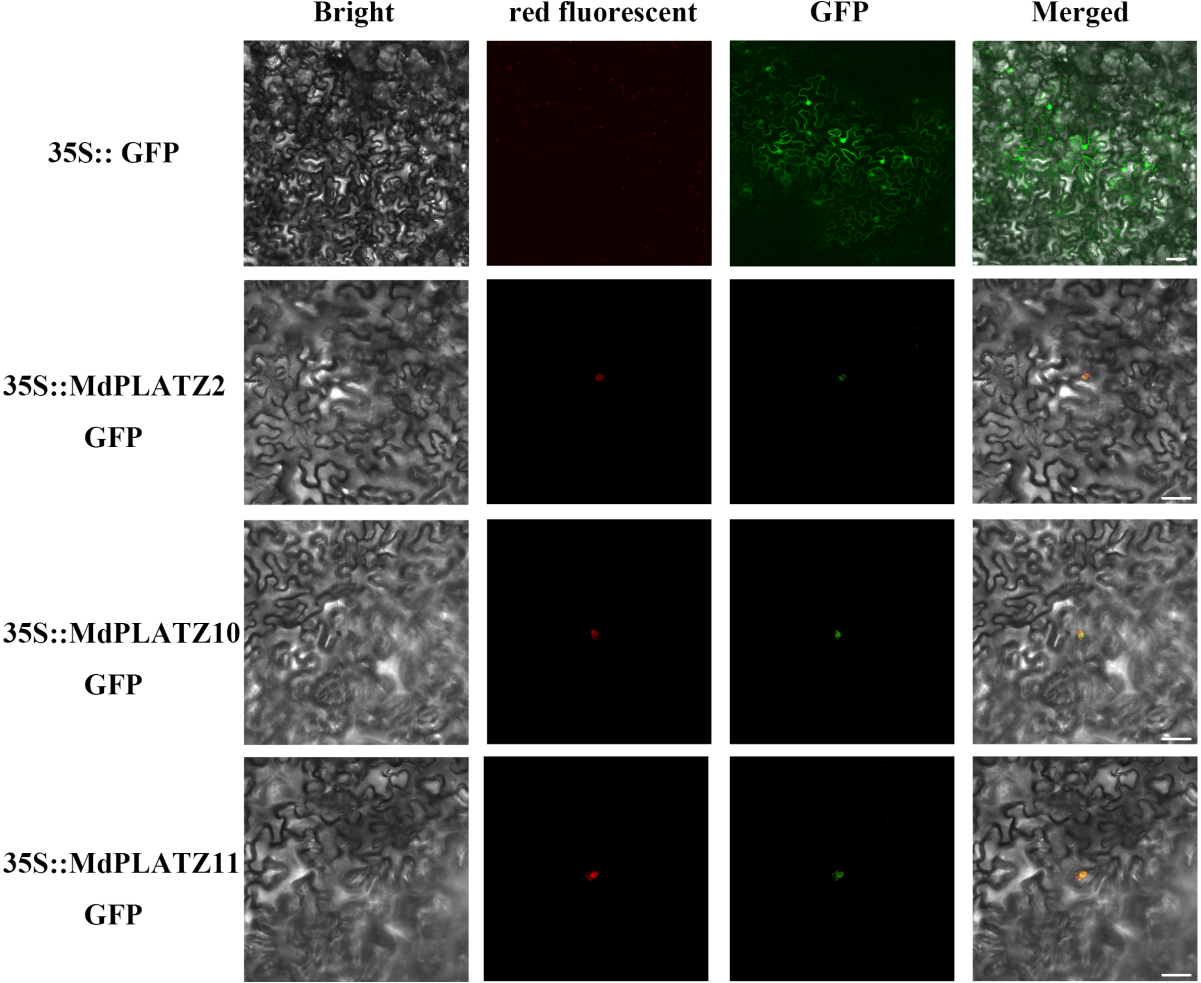
Subcellular localization of three MdPLATZ proteins. *35S::MdPLATZ2, 35S::MdPLATZ 10, 35S::MdPLATZ11::GFP*, pHBT-NLS-mCherry, and *35S::GFP* control were transiently expressed in *N. benthamiana* leaves and were detected under a confocal microscope. Scale bars represent 10 μm.

According to the prediction, most PLATZ genes in *M. domestica* were located in the nucleus (**Supplementary Table 1**). In order to better understand the characteristics of these proteins, three MdPLATZ genes with high expression levels in root under drought and ABA treatments, were selected for transient expression. As illustrated in **Figure 7**, the complete coding sequences (excluding the stop codons) of *MdPLATZ2*, *MdPLATZ10*, and *MdPLATZ11* were cloned into the pRI101-GFP vector under the control of the CaMV 35S promoter. The *N. benthamiana* leaves were used to transiently expressed a nuclear localization plasmid pHBT-NLS-mCherry. The GFP signals of *35S::MdPLATZ2/10/11::GFP* were observed in the nucleus and colocalization with the red fluorescence of NLS-mCherry protein in the nucleus. The signal of control vectors was detected in nucleus and cell membrane (**Figure 7**). These results suggested that three MdPLATZ proteins were located exclusively in the nucleus to participate in various biological processes.

### Gene co-expression network analysis of MdPLATZs

**Figure 8:**
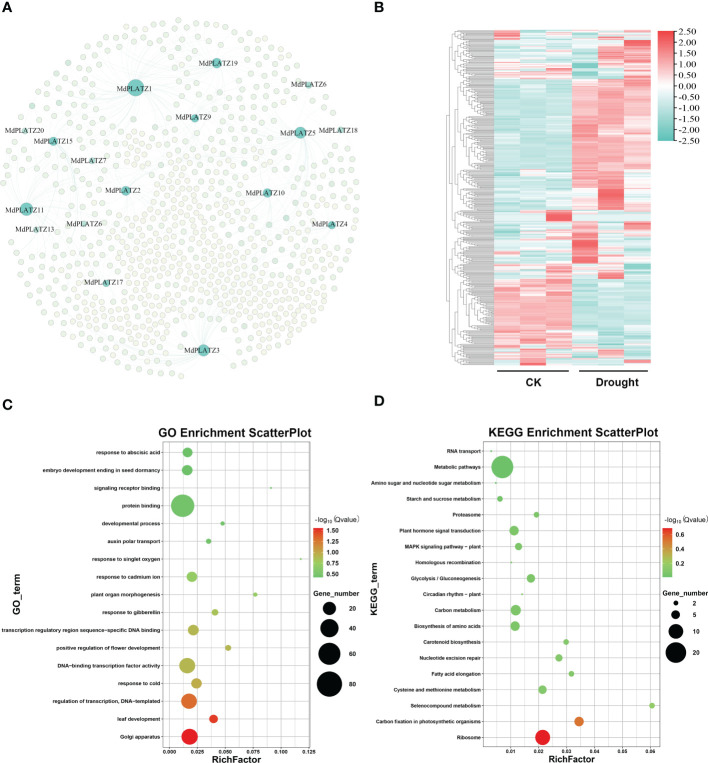
The co-expression network of *MdPLATZ* genes in *Malus*. **(A)** Co-expression network between 17 *MdPLATZ* and their co-expressed genes. The light green nodes indicate co-expressed genes, and the dark green nodes represent MdPLATZs. **(B)** Expressions of 412 PLATZ co-expressed genes in apple leaves from RNA-seq data under drought stress. **(C, D)** GO and KEGG enrichment analysis of the 412 co-expressed genes.

A co-expression network was constructed for PLATZ and their co-expressed genes (412 genes), predicted by AppleMDO database. The results showed that multiple genes, including *GRF9, KNOX*, and *NF-YA3*, might be regulated by MdPLATZs (**Figure 8A**; **Supplementary Table 4**). Subsequently, the expression patterns of MdPLATZ co-expression genes in apple leaves under drought stress were studied using public transcriptome data. As shown in **Figure 8B**, most co-expression genes were significantly up-regulated under drought stress, such as *DEAR3, AYHB15, bHLH79, GRF9, KNOX*, and *IBM1*, indicating that co-expression genes may play a vital role in response to drought stress (**Figure 8B**; **Supplementary Table 4**). Furthermore, the co-expression genes were functionally annotated using GO annotations. The result showed that the co-expression genes contained 17 GO categories, which belonged to cellular component, biological process and molecular function (**Figure 8C**). Obviously, multiple genes were annotated as “protein binding”, “transcription regulatory region sequence-specific DNA binding”, and “response to abscisic acid” categories (**Figure 8C**). In addition, the KEGG enrichment analysis results showed that multiple co-expression genes were enriched into “plant hormone signal transduction pathway” and “Metabolic pathways” (**Figure 8D**).

## Discussion

Rosaceae species (apples, pears, peaches, and strawberries, etc) are important economic fruit species worldwide, which provide unique contributions to healthy diet for human (Farinati et al., 2017). At present, drought stress is a great challenge to the growth and development of Rosaceae fruits, especially in apple. With the development of sequencing technology, a bunch of Rosaceae genomes had been completed, which laid a foundation for researchers to study the regulatory mechanisms of Rosaceae species in response to drought stress. PLATZ family genes are zinc-dependent DNA binding proteins, that had been proven to be involved in multiple biological processes in plants, including plant growth regulation, drought response, salt response, and leaf senescence regulation (Gonzalez-Morales et al., 2016; Li et al., 2021).

A total of 134 PLATZ genes were identified in the nine Rosaceae species genomes. The difference in the number of PLATZs genes among species suggested a gene duplication or deletion event occurred in Rosaceae species. Previous studies showed that an extra round of WGD event was shared in Maloideae (pear and apple) (Zhao et al., 2021a). The amount of PLATZ proteins in peach and strawberry were less than those in *Malus* and pear. Interestingly, the numbers of PLATZ proteins in cultivated apple species (Mdhf, Md, and Mdg) were less than those of the wild species (Msi, Msy, and Mb) within *Malus* species, indicating that the PLATZ genes might have occurred multiple loss or gain events during apple evolution. Tandem duplication and segmental duplication are the main driving force responsible for the expansion and diversification of gene families (Zhao et al., 2021b). Subsequently, the gene syntenic relationships and duplication events (tandem and segmental duplication) of PLATZ genes were analyzed in *Malus* species to investigate their evolutionary history. A total of 40 pairs of collinearity genes were detected in *Malus* PLATZ family genes, which were randomly distributed on nine to fourteen chromosomes. In addition, multiple segmental duplications and one tandem duplication were identified in *Malus*, indicating that segmental duplication is a major factor in the expansion of PLATZ in *Malus*. Similar results were also found in *Brassica rapa* and *Fagopyrum tataricum* (So et al., 2015; Azim et al., 2020).

In this study, the number of introns and exons of PLATZs were distinct among different groups, which might be related with their functional diversity. The transcriptome data showed that *MdPLATZ* genes expressed differentially in different tissues, which further supported that they might participate in different biological processes. Among them, seven *MdPLATZ* genes (*Mdplatz1*, *Mdplatz2*, *Mdplatz4*, *Mdplatz6*, *Mdplatz7*, *Mdplatz11*, and *Mdplatz19*) showed higher expression levels in roots, indicating that they might be involved in the root development, nutrient transport, or ion homeostasis. Previous study in *Arabidopsis* found that *AtPLATZ1* (orthologous of *Mdplatz1*) could regulate the growth of taproots under ABA treatment (Dong et al., 2021). *AtPLATZ3* (orthologous of *Mdplatz3*) was highly expressed in the leaves, and played a role in promoting leaf growth by accelerating cell proliferation (Jun et al., 2020). In addition, *AtPLATZ7* (orthologous of *Mdplatz7*) in *Arabidopsis* was preferentially expressed in the roots, which could control the size of the root meristem through ROS signals (Yamada et al., 2020). In this study, nine genes (*Mdplatz1*, *Mdplatz3*, *Mdplatz4*, *Mdplatz5*, *Mdplatz7*, *Mdplatz10*, *Mdplatz11*, *Mdplatz17*, and *Mdplatz20*) showed distinct expression patterns among buds and fruits collected from different developmental stages. These results indicated that those genes might be involved in the development of buds and fruits, which will be further investigated in the future studies.

Recently, increasing evidences have indicated that PLATZ genes are involved in drought stress (So et al., 2015; Zenda, 2019; Zhao et al., 2022). Drought stress induces the expression of *AtPLATZ4* in *Arabidopsis*, thereby affecting the expression of PR1, ABI3, ABI4 and ABI5 (Liu et al., 2020). In *Malus*, *Mdplatz2*, *Mdpaltz9*, *Mdpaltz10*, and *Mdplatz11* were also significantly induced under drought stress (**Figure 7**). These results indicate that the *MdPLATZ* genes played important roles in response to drought stress.

Gene co-expression network can provide useful evidence for determining gene regulation and attributing gene function to biological processes (Nayak et al., 2009; Rizwan et al., 2022). In this study, our co-expression network analyses revealed that the *MdPLATZ* genes were significantly correlated with multiple genes, including *NAC, bHLH79, GRF9*, and *NF-YA3*. Especially, most MdPLATZ co-expressed genes were significantly changed in apple under drought stress, such as *DEAR3, AYHB15*, *bHLH79, GRF9, KNOX*, and *IBM1*, which was consistent with previous studies. For example, *PbKNOX* gene was found to be highly expressed under drought stress, and was involved in plant growth and development in *Pyrus* (Liu et al., 2022). In *Camellia sinensis*, *CsbHLH79* was found to be positively correlated with drought and cold stresses (Samarina et al., 2020). *ZmNF-YA3* binds to the promoter of *bHLH92*, *FAMA*, and *MYC4* to improve drought tolerance in maize (Su et al., 2018). Furthermore, GO and KEGG enrichment analysis results showed that multiple co-expression genes were enriched into plant hormone signaling transduction pathway, which is in line with the fact that plants cope with various environmental stresses *via* regulating hormone synthesis and hormone signal transduction (Li et al., 2008; Xu et al., 2013; Zhang et al., 2022). Last but not least, a number of *MdPLATZ* genes were upregulated under ABA treatments in apple roots. Thus, we surmised that MdPLATZs and their co-expressed genes might be responsive to drought stress by mediating the ABA signaling pathway.

## Conclusion

The identification, structural and functional characterization of PLATZ genes were carried out in Rosaceae species with a highlight in *Malus*. Transcriptomic data showed that most *MdPLATZ* genes were extensively expressed in 16 apple tissues. Among them, *MdPLATZ2*, *MdPLATZ10*, and *MdPLATZ11* showed high expression levels in apple roots under drought and ABA treatments.

## Data availability statement

The original contributions presented in the study are included in the article/**Supplementary Material**. Further inquiries can be directed to the corresponding authors.

## Author contributions

YS designed the experiment, analysed the data and drafted the manuscript; YS, YL and JLia prepared the materials and performed the bioinformatic analysis; JLuo and FY did the qRT-PCR analysis; PF, HW, and BG assisted in editting the manuscript; FM and TZ conceived and designed the research. All authors contributed to the article and approved the submitted version.
