# Supplementary figures and images for: Identification of PLATZ genes in Malus and expression characteristics of MdPLATZs in response to drought and ABA stresses
Source: Front Plant Sci. 2023 Jan 18;13:1109784. doi: 10.3389/fpls.2022.1109784 (PMC9890193; doi:10.3389/fpls.2022.1109784)

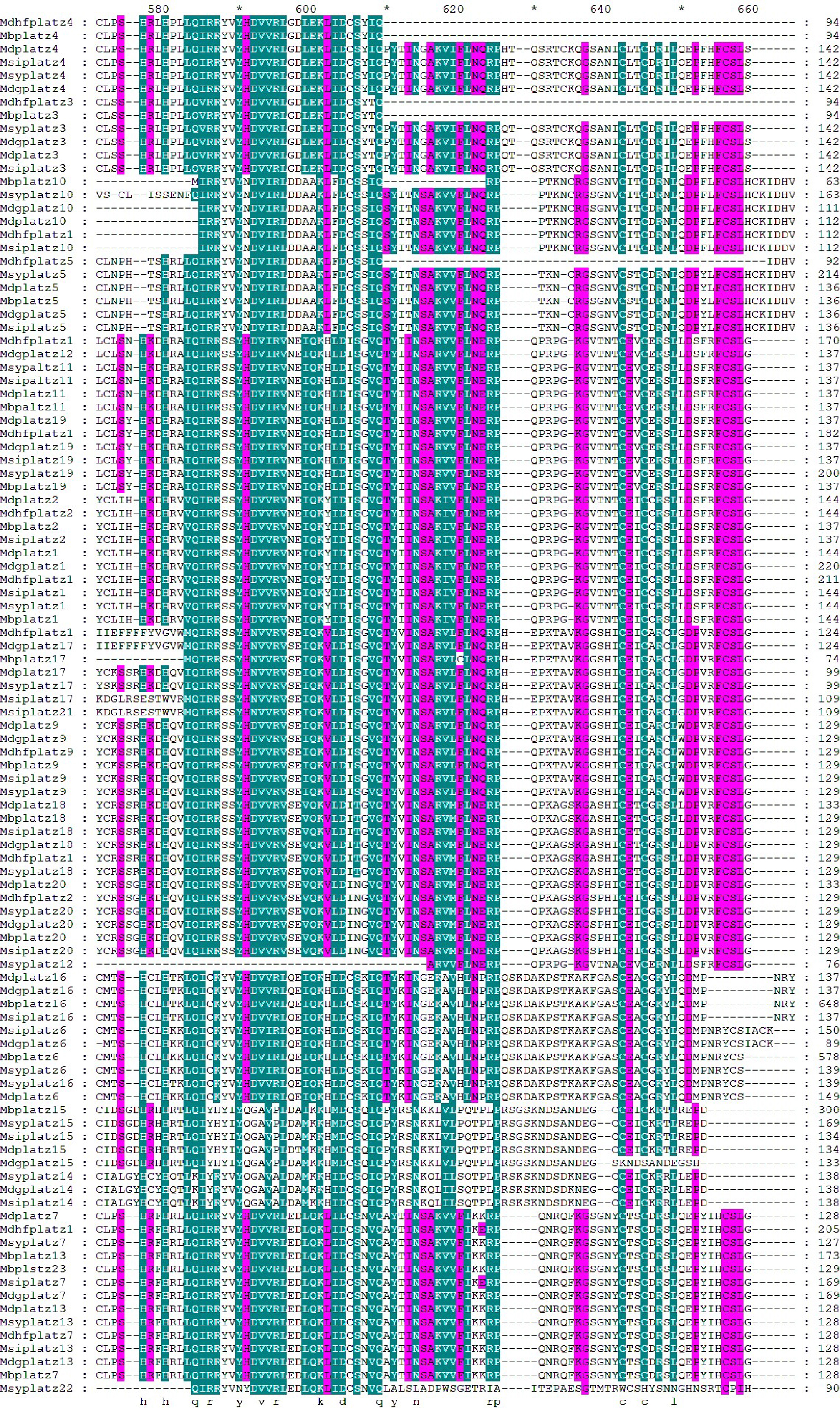

Supplement: Supplementary file 2 [file Image_1.jpeg]

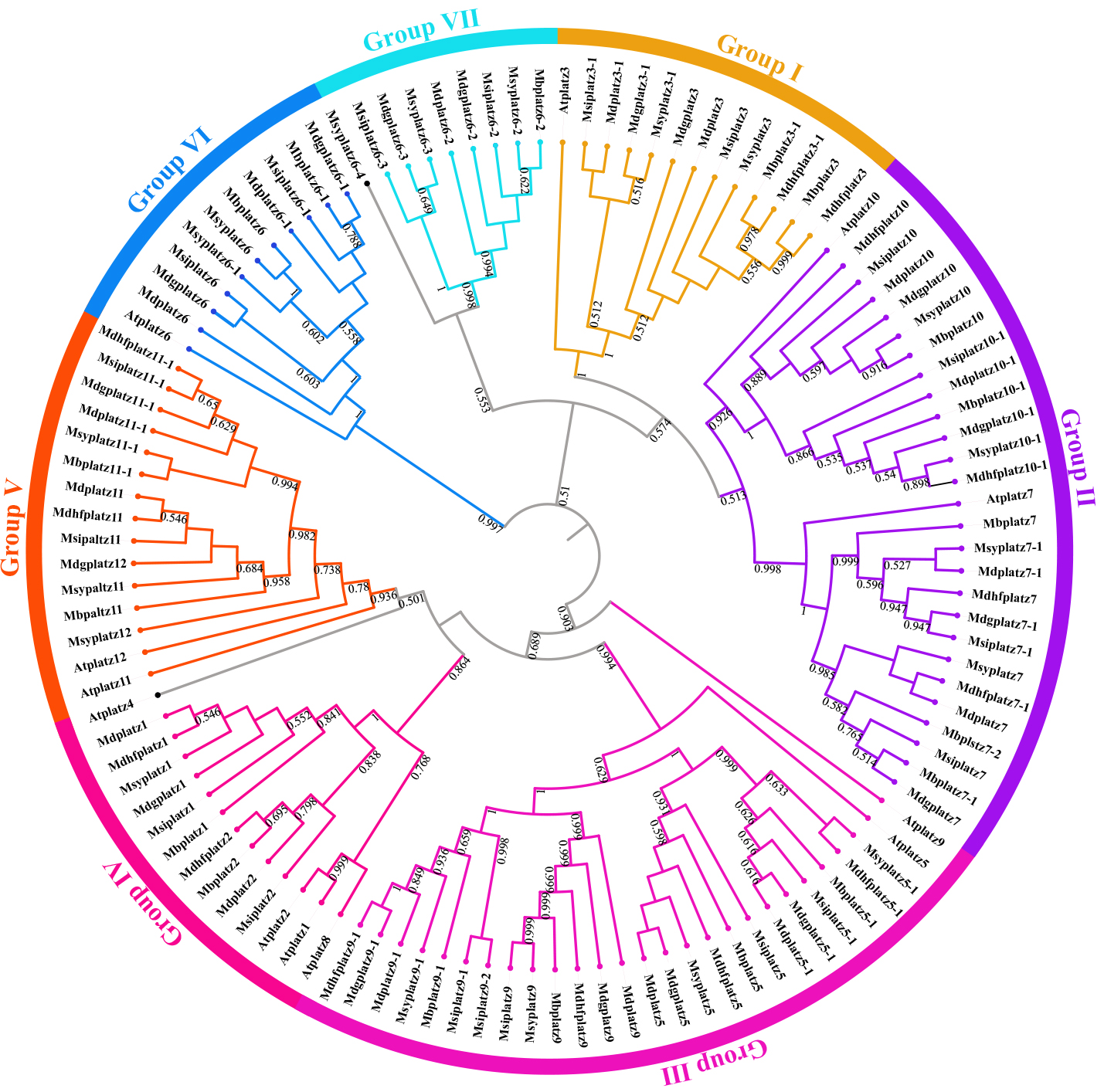

Supplement: Supplementary file 3 [file Image_2.jpeg]

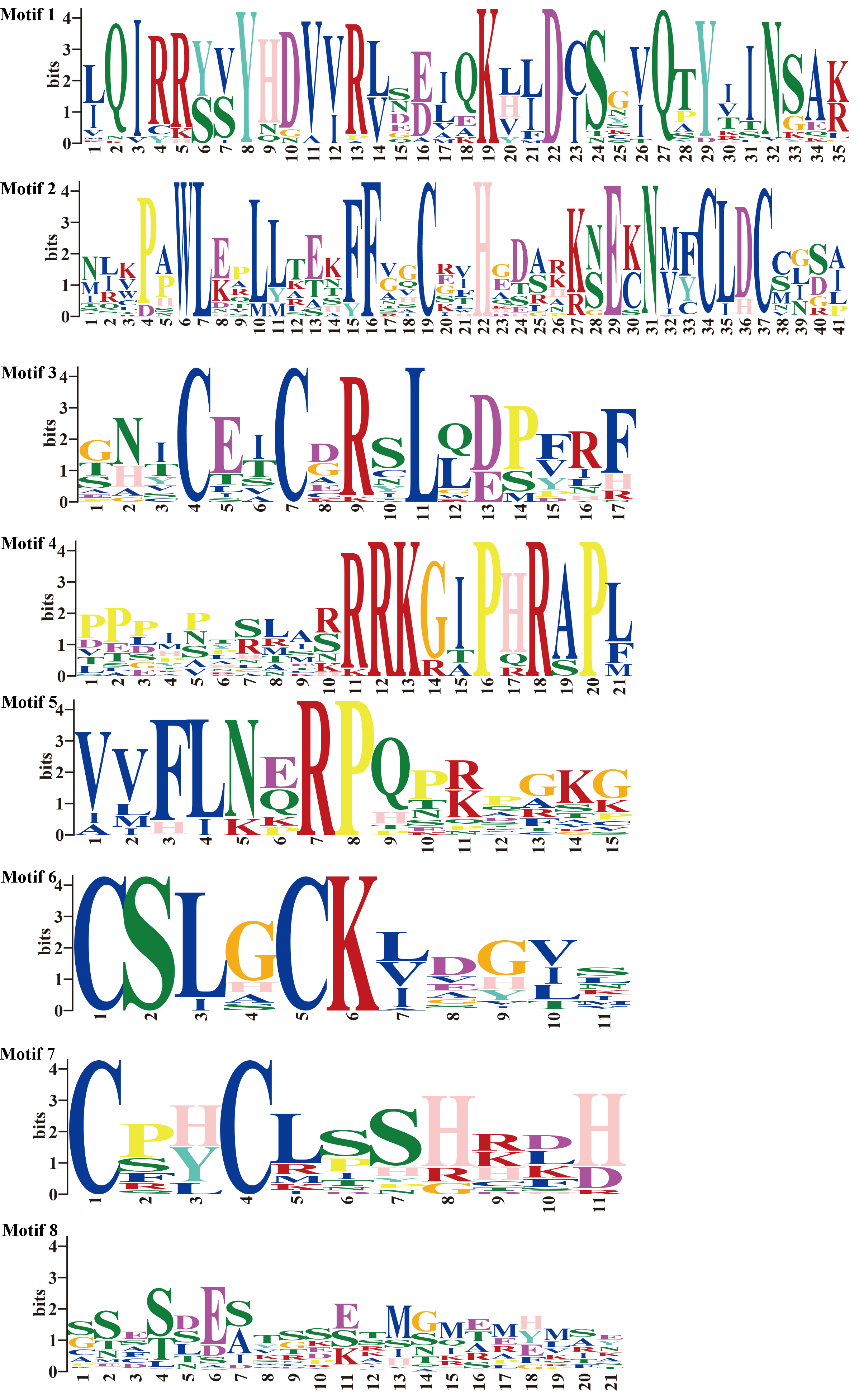

Supplement: Supplementary file 4 [file Image_3.jpeg]

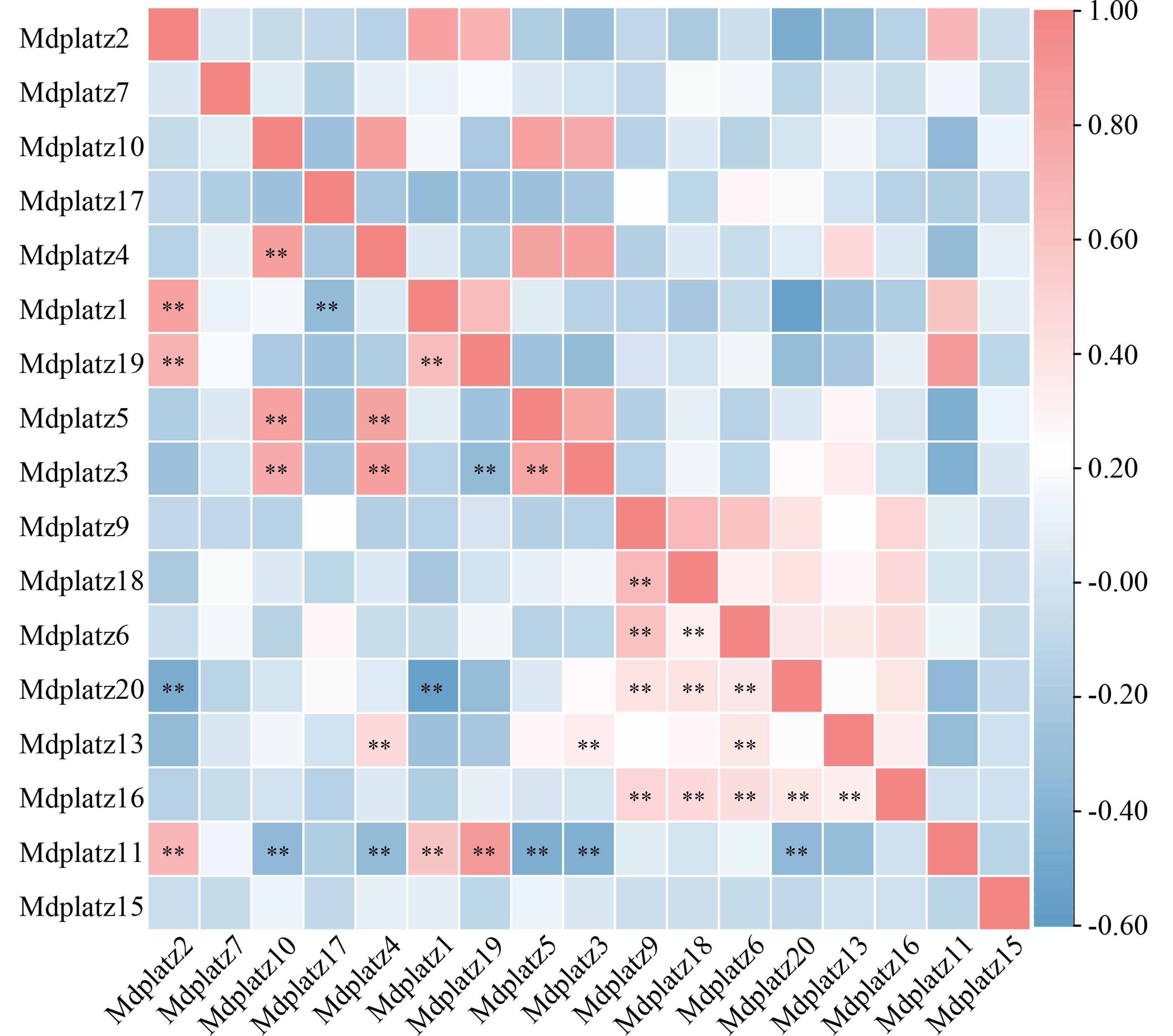

Supplement: Supplementary file 5 [file Image_4.jpeg]
